# Development of a Molecular-Subtype-Associated Immune Prognostic Signature That Can Be Recognized by MRI Radiomics Features in Bladder Cancer

**DOI:** 10.3390/bioengineering10030318

**Published:** 2023-03-02

**Authors:** Shenghua Liu, Haotian Chen, Zongtai Zheng, Yanyan He, Xudong Yao

**Affiliations:** 1 Department of Urology, Shanghai Tenth People’s Hospital, Tongji University, Shanghai 200072, China; 2 Urologic Cancer Institute, School of Medicine, Tongji University, Shanghai 200072, China; 3 Department of Pathology, Shanghai Tenth People’s Hospital, Tongji University, Shanghai 200072, China

**Keywords:** bladder cancer, molecular subtype, immune, prognostic signature, MRI, radiomics

## Abstract

**Background:** Bladder cancer (BLCA) is highly heterogeneous with distinct molecular subtypes. This research aimed to investigate the heterogeneity of different molecular subtypes from a tumor microenvironment perspective and develop a molecular-subtype-associated immune prognostic signature that can be recognized by MRI radiomics features. **Methods:** Individuals with BLCA in The Cancer Genome Atlas (TCGA) and IMvigor210 were classified into luminal and basal subtypes according to the UNC classification. The proportions of tumor-infiltrating immune cells (TIICs) were examined using The Cell Type Identification by Estimating Relative Subsets of RNA Transcripts algorithm. Immune-linked genes that were expressed differentially between luminal and basal subtypes and associated with prognosis were selected to develop the immune prognostic signature (IPS) and utilized for the classification of the selected individuals into low- and high-risk groups. Functional enrichment analysis (GSEA) was performed on the IPS. The data from RNA-sequencing and MRI images of 111 BLCA samples in our center were utilized to construct a least absolute shrinkage and selection operator (LASSO) model for the prediction of patients’ IPSs. **Results:** Half of the TIICs showed differential distributions between the luminal and basal subtypes. IPS was highly associated with molecular subtypes, critical immune checkpoint gene expression, prognoses, and immunotherapy response. The prognostic value of the IPS was further verified through several validation data sets (GSE32894, GSE31684, GSE13507, and GSE48277) and meta-analysis. GSEA revealed that some oncogenic pathways were co-enriched in the group at high risk. A novel performance of a LASSO model developed as per ten radiomics features was achieved in terms of IPS prediction in both the validation (area under the curve (AUC): 0.810) and the training (AUC: 0.839) sets. **Conclusions:** Dysregulation of TIICs contributed to the heterogeneity between the luminal and basal subtypes. The IPS can facilitate molecular subtyping, prognostic evaluation, and personalized immunotherapy. A LASSO model developed as per the MRI radiomics features can predict the IPSs of affected individuals.

## 1. Introduction

Globally, one of the most prevalent cancers related to urological cancer is bladder cancer [1]. Of newly diagnosed cases, 75% are cases of non-muscle-invasive bladder cancer (NMIBC), while 25% are cases of muscle-invasive bladder cancer (MIBC), the latter carrying a higher risk of disease progression and metastasis. Despite radical surgery and cisplatin-based chemotherapy, an immune checkpoint inhibitor has been introduced as a novel and safe method to treat BLCA [2]. Unfortunately, only nearly a quarter of patients benefit from immune checkpoint inhibitors, which highlights the importance of patient selection [2,3,4]. In recent years, molecular subtyping based on a comprehensive transcriptomic profile has been introduced to reflect the heterogeneity of bladder cancer, including UNC classification, Lund classification, MDA classification, and TCGA classification. Generally, these classifications overlap, and MIBC can be classified into luminal and basal subtypes [5,6,7,8]. Distinct intrinsic mechanisms and treatment responses among molecular subtypes have been identified [9,10], but the heterogeneity of different molecular subtypes has not been elucidated from an immunological perspective.

The tumor microenvironment (TME), a complicated system, includes diverse components that have important roles in immunotherapy response and prognosis [11,12]. As a crucial component of TME, tumor-infiltrating immune cells (TIICs) reflect the status of TME and perform vital functions in inhibiting and promoting tumor growth and progression [13]. In addition, some TIICs, such as regulatory T cells and macrophages, have a strong association with immune escape, thereby leading to the failure of immunotherapy [14].

Magnetic resonance imaging (MRI) is preferred and recommended for preoperative diagnosis of BLCA in clinical practice. MRI images can be examined through computational medical imaging, also called radiomics, by extracting high-throughput quantitative features that cannot be deciphered by the human eye, thus allowing preoperative prediction of the biological behavior at tumor onset through a non-invasive approach [15,16]. In addition, prior research has linked features extracted from CT images to gene expression patterns in bladder cancer [17,18]. Therefore, it could be assumed that radiomics could be used to construct a non-invasive, convenient, and efficient model for genetic status prediction.

This research aimed to elucidate the different mechanisms underlying the link between luminal and basal subtypes from an immunological perspective in BLCA. MIBC was classified into luminal and basal subtypes according to the UNC classification system. The Cell Type Identification by Estimating Relative Subsets of RNA Transcripts (CIBERSORT) algorithm was applied to present a TIIC landscape and explore the differences in TIICs between the subtypes. As per the expression of five immune-related genes (IRGs), an immune prognostic signature (IPS) was developed which was shown to have a high association with molecular subtypes and critical immune checkpoint gene expression. The IPS exhibited good performance in predicting prognosis and immunotherapy response. An IPS-based nomogram was constructed for prognostic prediction. Furthermore, RNA-sequencing and preoperative MRI data of 111 individuals with BLCA in our center were utilized to construct a least absolute shrinkage and selection operator (LASSO) model for IPS prediction.

## 2. Materials and Methods

### 2.1. Data Collection

**Figure 1:**
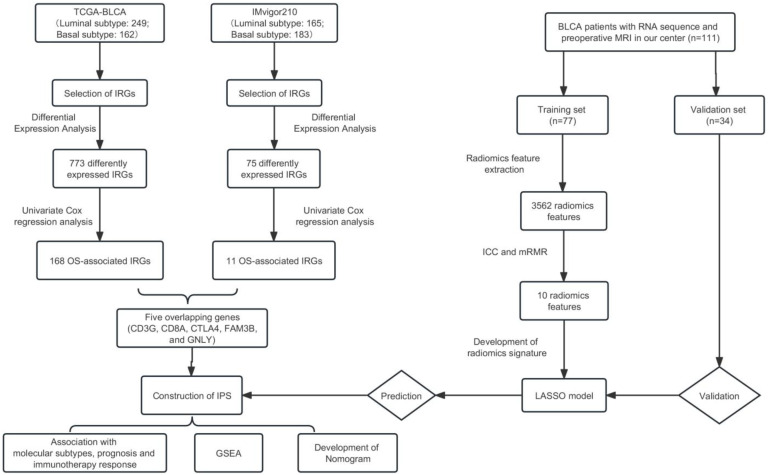
The workflow of this study. TCGA, The Cancer Genome Atlas; BLCA, bladder cancer; IRGs, immune-related genes; OS, overall survival; IPS, immune prognostic signature; GSEA, functional enrichment analysis; ICC, intra- and interclass correlation coefficient; mRMR, minimum redundancy maximum relevance; LASSO, least absolute shrinkage and selection operator.

Figure 1 shows the workflow of the present study.

Individuals with BLCA in Shanghai Tenth People’s Hospital were subject to the following inclusion criteria: (1) histologically diagnosed as BLCA; (2) samples were accessible for RNA-sequencing after surgery; (3) MRI was performed within 20 days before surgery. The criteria for exclusion were as follows: (1) any treatments were performed before MRI examination; (2) poor-quality MRI images; (3) tumors with poorly defined boundaries; (4) the subjects lacked baseline clinical factors. RNA sequencing, paired-end library generation, total RNA extraction, and MRI examination were performed as per the prior description [19,20]. Random classification of BLCA subjects into the training and validation sets at a 7:3 ratio was performed.

The standardized expression data set of mRNA (RNA-sequence) Fragments Per Kilobase of transcript per Million Fragments along with clinical data were retrieved from The Cancer Genome Atlas (TCGA)-BLCA cohort (https://portal.gdc.cancer.gov, accessed on 25 September 2022). The IMvigor210 trial provided data on atezolizumab (PD-L1 inhibitor)-treated subjects (metastatic urothelial cancer subjects), such as clinical and RNA expression data (http://research-pub.gene.com/IMvigor210CoreBiologies, accessed on 25 September 2022). The Gene Expression Omnibus (GEO) provided the RNA expression data for GSE32894, GSE31684, GSE13507, and GSE48277, along with their corresponding prognostic data (https://www.ncbi.nlm.nih.gov/geo/, accessed on 25 September 2022). The average value of RNA expression was calculated for gene symbols with multiple probes.

### 2.2. Evaluation of TIICs

As per the marker gene’s RNA expression data, the CIBERSORT algorithm was run to assess 22 TIICs in terms of their relative proportions (https://cibersort.stanford.edu, accessed on 25 September 2022). Monte Carlo sampling was applied for the deconvolution and CIBERSORT *p*-value calculation for every sample. The CIBERSORT *p*-value < 0.05 was set as the sample selection threshold for examining the proportions of TIICs. The TCGA data set, which had the highest number of MIBC subjects, was utilized for assessing various TIICs.

### 2.3. RNA Expression Data-Based Molecular Subtyping

The molecular subtypes of subjects with MIBC were obtained using the ‘BLCA subtyping’ R package (https://github.com/cit-bioinfo/BLCAsubtyping, accessed on 25 September 2022) based on the RNA expression data. Six classification approaches, including UNC, CIT, Lund (2017), MDA, Baylor, and TCGA classifications (2017), were applied for molecular subtyping.

### 2.4. Molecular-Subtype-Associated IRGs

The IRG list was accessed from the Immunology Database and Analysis Portal database (https://immport.niaid.nih.gov, accessed on 25 September 2022). The TCGA and IMvigor210 patients were divided into luminal and basal subtypes as per the UNC classification. The differentially expressed IRGs (adjusted *p*-value < 0.05 and |Fold change| > 0.5) between luminal subtypes and basal subtypes were obtained using the ‘limma’ R package v3.46 in TCGA and IMvigor210, respectively. The resulting IRGs were analyzed through univariate Cox regression to further identify the overall survival (OS)-associated IRGs in TCGA and IMvigor210, respectively. The OS-associated IRGs were then imported into an online Venn diagram tool (http://bioinformatics.psb.ugent.be/webtools/Venn/, accessed on 25 September 2022) to find the overlapping genes between TCGA and IMvigor210.

### 2.5. IPS Construction and Validation

The IPS was developed per the expression of the overlapping genes between TCGA and IMvigor210. The formula for the IPS is presented below:

Metabolic score=scale (∑X−∑Y)

where *X* and *Y* represent the selected gene with HR > 1 and HR < 1, respectively. The formula above was utilized for quantifying the patient’s risk score. The R package ‘survminer’ v0.4.8 calculated the optimal threshold values, according to which high- and low-risk groups of the subjects were established. The prognosis predictive performance of the IPS was investigated using the Kaplan–Meier and log-rank tests in TCGA and IMvigor210. Four GEO data sets (GSE32894, GSE31684, GSE13507, and GSE48277) were used to further validate the prognosis predictive potential of the IPS.

Additionally, a meta-analysis was performed utilizing the results of the univariate Cox regression analyses (confidence interval (CI) of 95% and HR) for each data set. The heterogeneity of the meta-analysis was evaluated using the *I*^2^ statistic and the χ^2^-based Q test and was assessed by the *I*^2^ value. The fixed-effect inverse-variance model was utilized when the *I*^2^ value was < 25% and the *p*-value was > 0.05 and the heterogeneity was considered to be low. Any possible publication bias was assessed by means of Egger’s test and Begg’s plotting, where bias was considered to be absent at a *p*-value > 0.05.

### 2.6. Gene Set Enrichment Analysis (GSEA)

The biological processes between high- and low-risk values were compared using GSEA software (http://www.broad.mit.edu/GSEA/, accessed on 25 September 2022) in terms of their different biological pathways (http://www.broadinstitute.org/gsea/index.jsp, accessed on 25 September 2022). GSEA is able to uncover biological processes based on sets of differentially expressed genes instead of individual genes. Significant results were defined as a false discovery rate < 0.25 and a nominal *p*-value < 0.05.

### 2.7. Nomogram Construction

Univariate and multivariate Cox regression analyses were utilized to investigate the prognostic significance of clinicopathological features and IPS in TCGA. The significant variables in multivariate Cox regression analysis were selected for nomogram construction. The nomogram’s performance was examined through calibration plots that utilized the ‘Rms’ R package v5.1. The potential of the nomogram in clinical settings was investigated through decision curve analysis (DCA).

### 2.8. MRI Protocal

Patients were instructed to drink water 1 h before the MRI study and to present with a full bladder. Imaging was performed with a 3-T MRI system (Magnetom Trio; Siemens Healthcare, Munich, Germany) using an eight-channel phased-array pelvic coil. Conventional T1-weighted spin-echo images (499 ms repetition time (TR), 12 ms echo time (TE); 204 × 256 matrix; 40 cm field of view; 5 mm section thickness; 1.3 mm intersection gap; two signals acquired) and T2-weighted turbo spin-echo images (4000 ms TR, 100 ms TE; 256 × 256 matrix; 20 cm field of view; 5 mm section thickness; 1 mm intersection gap; two signals acquired) were obtained in the axial plane. High-resolution T2 weighted turbo spin-echo images (4700 ms TR, 107 ms TE; 256_256 matrix; 16 cm field of view; 5 mm section thickness; 1 mm intersection gap; one signal acquired) were obtained in three orthogonal planes. DW images were obtained using a single-shot spinecho echoplanar sequence (b, 0 and 1500 sec/mm^2^ (DW gradients applied in three orthogonal directions); TR/TE,5200/74.9 ms; matrix, 96 × 130; section thickness, 3 mm; gap, 1 mm; field of view, 28 × 28 cm; number of sections, 27; NEX, 6).

Contrast-enhanced MRI was performed in the axial plane using 2D turbo FLASH sequences with an IV bolus administration by hand injection of 0.1 mmol/kg of body weight of a gadolinium chelate contrast agent. In the following contrast sequences, 10 turbo FLASH series, each lasting 30 s, were performed sequentially in 5 min using parameters identical to those of the unenhanced sequence. The onset of the contrast injection and the data acquisition were triggered synchronously.

### 2.9. Region of Interest (ROI) Segmentation and Feature Extraction

An open-source, free software package (ITK-SNAP, v3.6.0; http://itk-snap.org) was utilized by a radiologist (F Xu, experienced in bladder MRI reading with more than 5 years of practice) to manually delineate the ROIs along the edges of the tumors, slice by slice, on T2WI images and the delay phase of dynamic contrast-enhanced (DCE) images. In the case of numerous lesions in an individual with BLCA, only the biggest lesion was analyzed further. In order to examine the reliability of the observer, another radiologist was selected to perform the same experiment. The same radiologist performed his experiment after a month utilizing randomly selected individuals that were 40 in number alongside another new radiologist (T Xu). The reliability of their observations was examined by assessing the interobserver reliability.

Four types of radiomics features, including image intensity (first-order features), shape- and size-based, wavelet, and textural features, were obtained from the PyRadiomics platform (http://www.radiomics.io/pyradiomics.html, accessed on 25 September 2022) [21]. The ROIs of each subject were utilized for this extraction. Ultimately, the extraction of 3562 radiomics features from T2WI and DCE images (1781 radiomics features from T2WI and DCE images, respectively) was carried out with subsequent normalization of each feature with a Z-score prior to feature selection.

### 2.10. Feature Selection and Radiomics Signature Development

To examine the interobserver reliability, inter- and intraclass correlation coefficients (ICCs) were derived. Features with ICCs > 0.75 were selected for the minimum redundancy maximum relevance (mRMR). mRMR is a supervised feature-selection algorithm which calculates the mutual information (MI) between a target variable and features. It ranks features by maximizing MI with respect to the target variable and then minimizes the average MI for features with higher rankings [22]. The importance of features was ranked through the mRMR algorithm, and the 10 leading features were selected for radiomics signature development. In this study, the LASSO algorithm was used via the R package “glmnet” for radiomics signature development. The LASSO algorithm can select features with non-zero coefficients and remove features with negligible effect on the target variable, which is able to prevent overfitting and enhance model interpretation [23]. The penalty regularization parameter lambda (λ) was chosen via 10-fold cross-validation to obtain radiomics features with non-zero coefficients and minimize the mean square error. Meanwhile, the minimal λ was identified to obtain the radiomics features. Each subject’s radiomics score was calculated as follows:

$$\mathrm{Radiomics}\;\mathrm{score}=\sum_{i=1}^{n}Coef_{i}\times x_{i}$$

where *x_i_* is the standardized value of each selected radiomics feature and *Coef_i_* is the coefficient corresponding to each radiomics feature.

The optimal parameter configuration obtained from the training set was utilized for the construction of the radiomics signature, which was subsequently evaluated in the validation set. The performance of the above-mentioned signature was evaluated through the quantification of the specificity, sensitivity, accuracy, negative and positive predictive values (NPV and PPV, respectively), and the area under the receiver operating characteristic (ROC) curve (AUC).

### 2.11. Statistical Analysis

One-way ANOVA, Wilcoxon testing, or *t*-testing was utilized for the variable evaluation of the subjects categorized by molecular subtypes and risk groups. The ‘Pheatmap’ R package v1.0.12 and the ‘ggalluvial’ R package v0.12.3 were utilized to generate heatmap and alluvial diagrams, respectively. R v3.6.1 (https://www.r-project.org/, accessed on 25 September 2022) and SPSS 23.0 (SPSS, Armonk, NY, USA) were employed to analyze the data statistically. The meta-analyses were performed using the R package ‘meta’ v4.16 (Aaron Lun, San Francisco, CA, USA) and the R package ‘metafor’ v2.4 (Aaron Lun, San Francisco, CA, USA). The link of IPS to the expression of critical immune checkpoint genes was explored using the ‘corplot’ R package v4.0.3 (Alexander Ploner, Stockholm, Sweden). Significant results were defined as a two-sided *p*-value < 0.05.

## 3. Results

### 3.1. Comparison of TIICs between Luminal and Basal Subtypes

**Figure 2:**
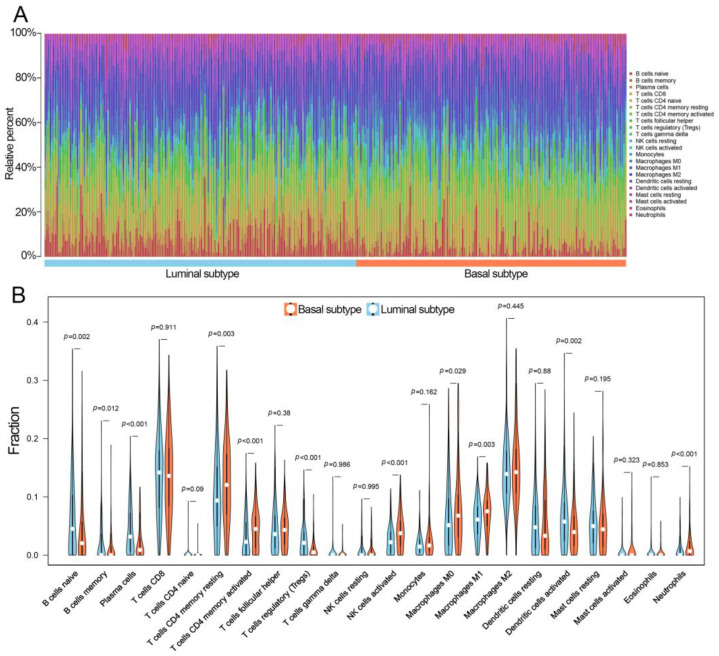
The TIICs of MIBC in TCGA. (**A**) The distributions of 22 TIICs in each MIBC sample evaluated by the CIBERSORT algorithm. (**B**) The differences in TIICs between luminal and basal subtypes. MIBC, muscle-invasive bladder cancer; TIICs, tumor-infiltrating immune cells; CIBERSORT, Cell Type Identification by Estimating Relative Subsets of RNA Transcripts.

The percentages of 22 TIICs in 343 MIBC subjects (including 185 luminal and 158 basal subtypes) in TCGA with CIBERSORT *p* < 0.05 were examined (Figure 2A). There were 11 out of 22 TIICs that showed a difference in distribution between luminal and basal subtypes. Specifically, the basal subtypes had relatively higher percentages of Macrophages M0, Macrophages M1, NK cells activated, resting memory, and memory-activated CD4 T-cells, as well as neutrophils. Conversely, the luminal subtypes had higher percentages of dendritic cells activated, regulatory T cells (Tregs), plasma cells, memory B cells, and naïve B cells (Figure 2B).

### 3.2. Selection of IRGs

**Figure 3:**
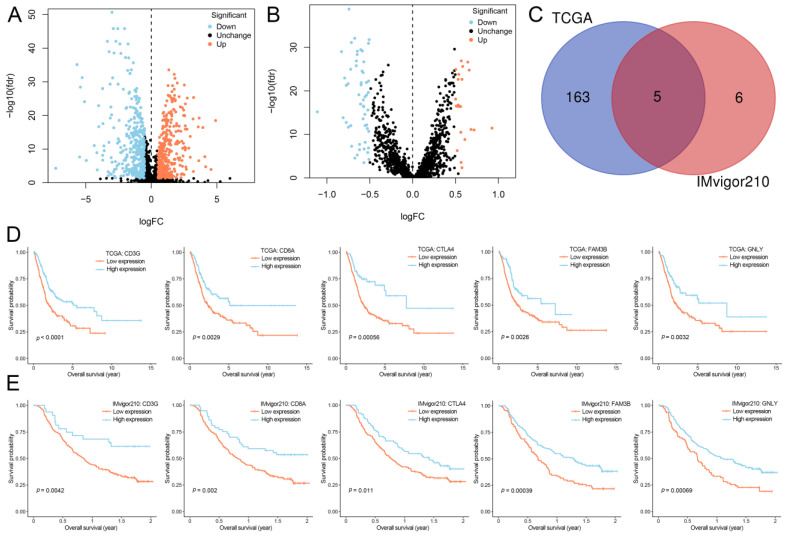
Selection of OS-associated IRGs. (**A**) Volcano plot of 739 IRGs differentially expressed between luminal and basal subtypes in TCGA. (**B**) Volcano plot of 75 IRGs differentially expressed between luminal and basal subtypes in IMvigor210. (**C**) Venn diagram of the five common OS-associated IRGs between TCGA and IMvigor210. (**D**) Kaplan–Meier curves of OS for patients based on five selected IRGs in TCGA. (**E**) Kaplan–Meier curves of OS for patients based on five selected IRGs in IMvigor210. OS, overall survival; IRGs, immune-related genes; TCGA, The Cancer Genome Atlas; FC, fold change.

According to the UNC classification system, individuals in TCGA and IMvigor210 were classified into luminal (249 and 165, respectively) and basal subtypes (162 and 183, respectively). There were 739 and 75 differentially expressed IRGs in TCGA and IMvigor210, respectively (Figure 3A,B). After the univariate Cox regression analysis, the OS-associated IRGs in TCGA and IMvigor210 were 168 and 11, respectively. The Venn diagram showed that there were five overlapping genes (CD3G, CD8A, CTLA4, FAM3B, and GNLY) between TCGA and IMvigor210 (Figure 3C). The survival analyses of five genes were investigated (Figure 3D,E).

### 3.3. Construction and Performance of the IPS

**Figure 4:**
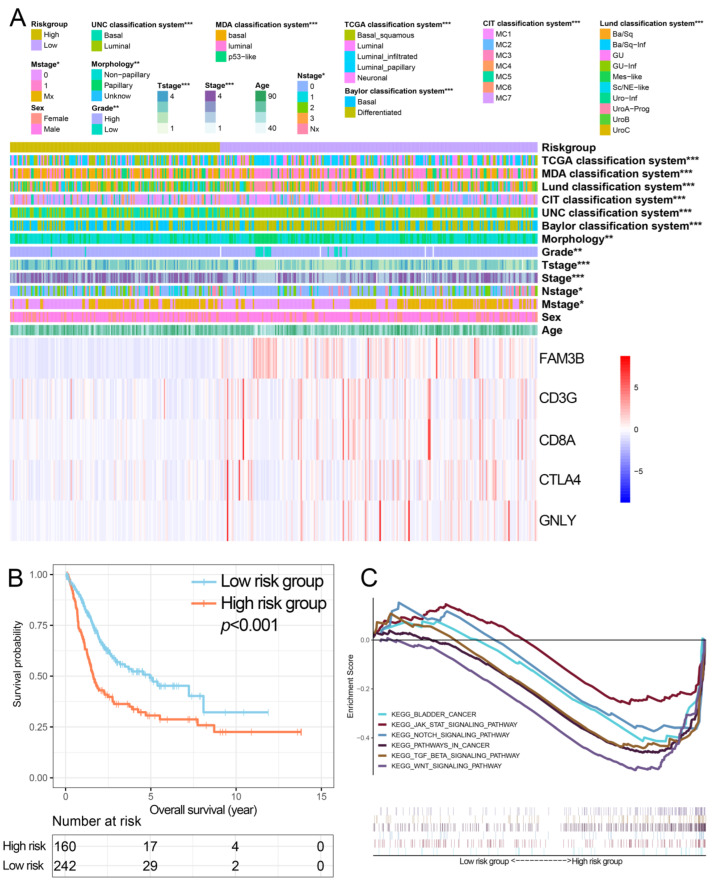
Performance of the IPS in TCGA. (**A**) The distribution of molecular subtypes and clinicopathological features were compared between low- and high-risk groups. (**B**) Kaplan–Meier curves of overall survival for patients based on IPS. (**C**) Gene set enrichment analysis revealed significant oncogenic pathways associated with IPS. IPS, immune prognostic signature; TCGA, The Cancer Genome Atlas; KEGG, Kyoto Encyclopedia of Genes and Genomes. * *p* < 0.05, ** *p* < 0.01, *** *p* < 0.001.

Each subject’s risk score was derived based on the expression of the five genes and the aforementioned formula. In TCGA, the high-risk group was positively associated with clinicopathological features, including pathological stage, morphology, histological grade, as well as the T, M, and N stages (Figure 4A), and subjects in this group had considerably poorer OS than those at lower risk (Figure 4B). GSEA showed that several oncogenic KEGG signaling pathways, including ‘BLADDER_CANCER’, ‘JAK_STAT’, ‘NOTCH, ‘KEGG_PATHWAYS_IN_CANCER’, ‘TGF_BETA’, and ‘WNT’, were coenriched in the more at-risk group (Figure 4C).

**Figure 5:**
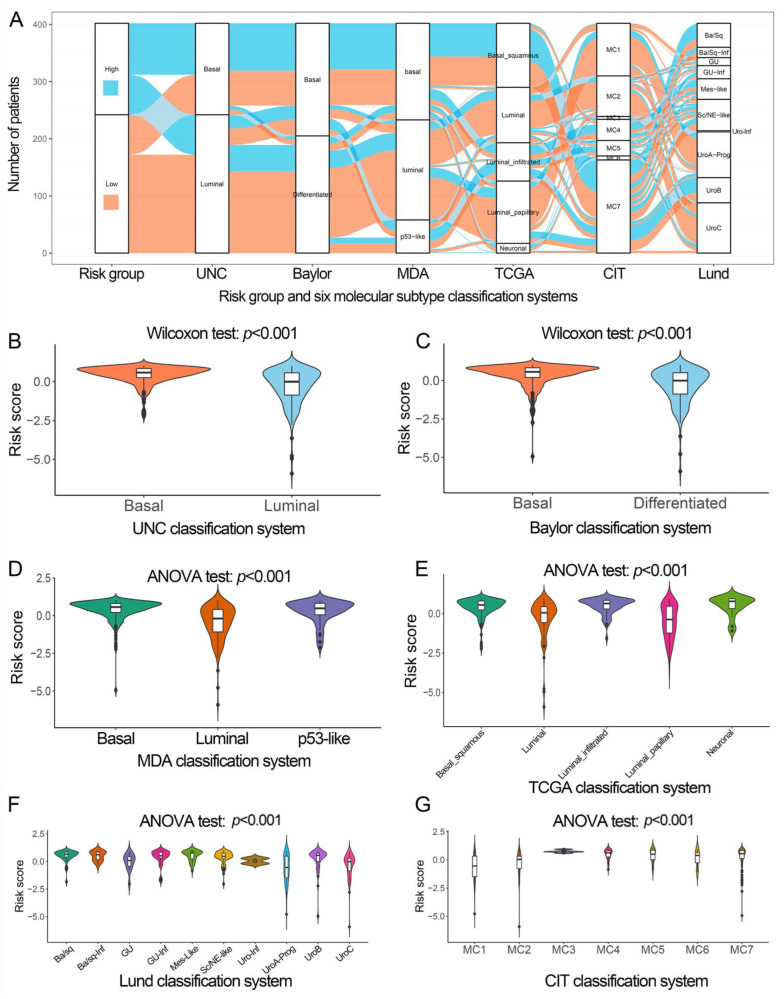
The relationship between IPS and molecular subtypes of different classification systems in TCGA. (**A**) Alluvial diagram presents the changes in risk groups and indicates the molecular subtypes of different classification systems. (**B**–**G**) Differences in IPS between molecular subtypes in UNC, Baylor, IPS, MDA, TCGA, Lund, and CIT classification systems. IPS, immune prognostic signature; TCGA, The Cancer Genome Atlas.

The risk group was considerably linked to six classification approaches (Figure 5A), and the basal subtype had a significantly higher risk score than the luminal subtype (Figure 5B–G).

**Figure 6:**
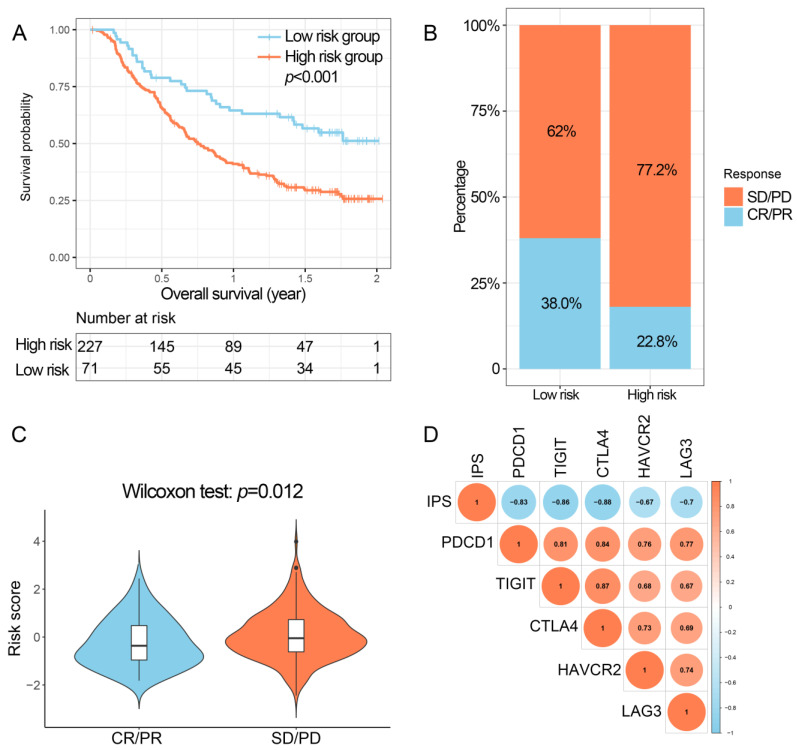
Performance of the IPS in IMvigor210. (**A**) Kaplan–Meier curves of overall survival for patients based on IPS. (**B**) Differences in immunotherapy response between low- and high-risk groups. (**C**) Differences in IPS between CR/PR and SD/PD subgroups. (**D**) The correlation between IPS and expression of critical immune checkpoints. IPS, immune prognostic signature; CR, complete response; PR, partial response; SD, stable disease; PD, progressive disease.

In IMvigor210, subjects in the low-risk group had poorer OS than those in the high-risk group (Figure 6A). In addition, subjects in the low-risk group had an increased percentage of partial response (PR)/complete response (CR) compared to those in the high-risk group (38.0% and 22.8%, respectively; Figure 6B). PR/CR subjects had considerably decreased risk scores compared to progressive disease (PD)/stable disease (SD) subjects (Figure 6C). In addition, IPS was negatively related to the expression profiles of critical immune checkpoint genes (Figure 6D).

### 3.4. Validation of the IPS

**Figure 7:**
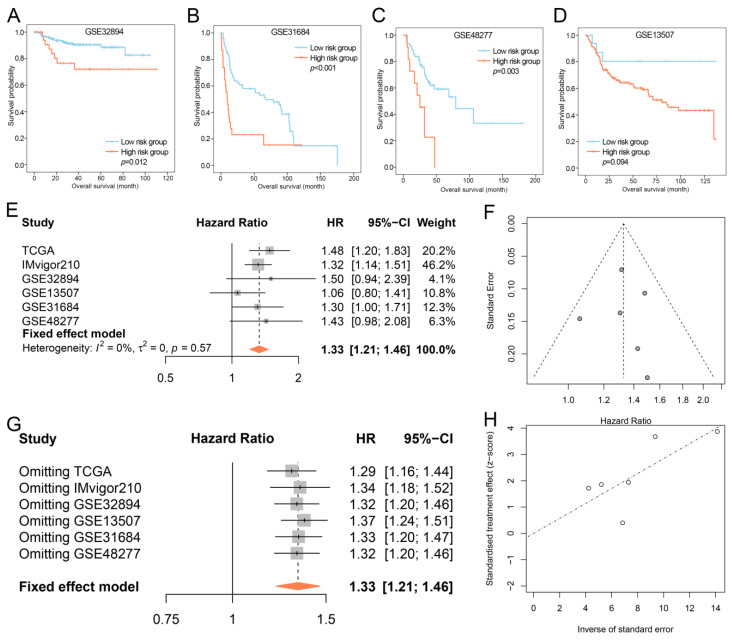
Prognostic value of IPS in validation data sets. (**A**–**D**) Kaplan–Meier OS curves for patients assigned to high- and low-risk groups in the GSE32894 (**A**), GSE31684 (**B**), GSE48277 (**C**), and GSE13507 (**D**) data sets. (**E**) Forest plots of pooled HRs for investigating the impact of IPS on OS in each data set. (**F**) Funnel plots of meta-analysis. (**G**) Sensitivity analysis of meta-analysis. (**H**) Egger’s publication bias plot of meta-analysis. IPS, immune prognostic signature; OS, overall survival; HR, hazard ratio; CI, confidence interval.

Except for GSE13507, IPS showed potential for prognosis prediction in GSE32894, GSE31684, and GSE48277 through Kaplan–Meier and log-rank tests (Figure 7A–D). Meta-analysis was further utilized for a comprehensive investigation of the prognostic value of IPS. As the heterogeneity test revealed negligible heterogeneity among data sets (I^2^ = 0.00%, *p*-value = 0.53), the fixed-effect inverse-variance model was used for the meta-analysis. The results suggested that for individuals suffering from BLCA, IPS could act as a risk factor (Figure 7E,G; *p* < 0.001, HR = 1.31, 95% CI: 1.18–1.43). The funnel plot was also basically symmetrical (Figure 7F). The Egger’s test (*p* = 0.935), Begg’s test (*p* = 0.719), and Egger’s publication bias plot revealed no publication bias (Figure 7H).

### 3.5. Construction of Nomogram

**Figure 8:**
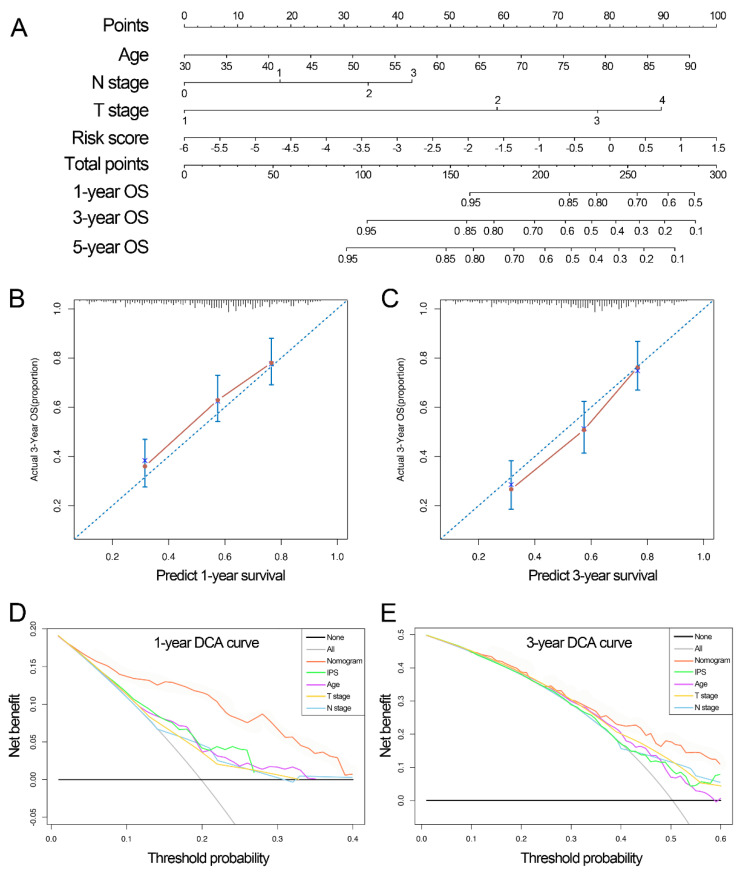
Development of the IPS-based nomogram in TCGA. (**A**) The IPS−based nomogram for predicting the probability of 1−, 3−, and 5−year OS. (**B**,**C**) Calibration plots of the nomogram for predicting the probability of 1− and 3−year OS. (**D**,**E**) DCA of the nomogram for 1− and 3−year OS. IPS, immune prognostic signature; TCGA, The Cancer Genome Atlas; OS, overall survival; DCA, decision curve analysis.

In a univariate Cox regression analysis, IPS, age, monograph, stage, histological grade, T stage, and N stage were linked to OS and introduced into multivariate Cox regression analysis (Table 1). IPS, age, and T and N stages were indicated to be significant and were selected to develop the nomogram for predicting 1-, 3- and 5-year OS (Figure 8A). Calibration plots presented a considerable consistency with actual 1- and 3-year OS (Figure 8B,C). DCA further indicated the clinical usefulness of the nomogram (Figure 8D,E).

### 3.6. Radiomics Signature Development and Performance Determination

This research examined the data of 111 BLCA subjects. The training and validation sets comprised 77 and 34 subjects, respectively. Data regarding the clinical features of the subjects were assessed (Table 2). The two sets did not exhibit any differences that were statistically significant.

**Figure 9:**
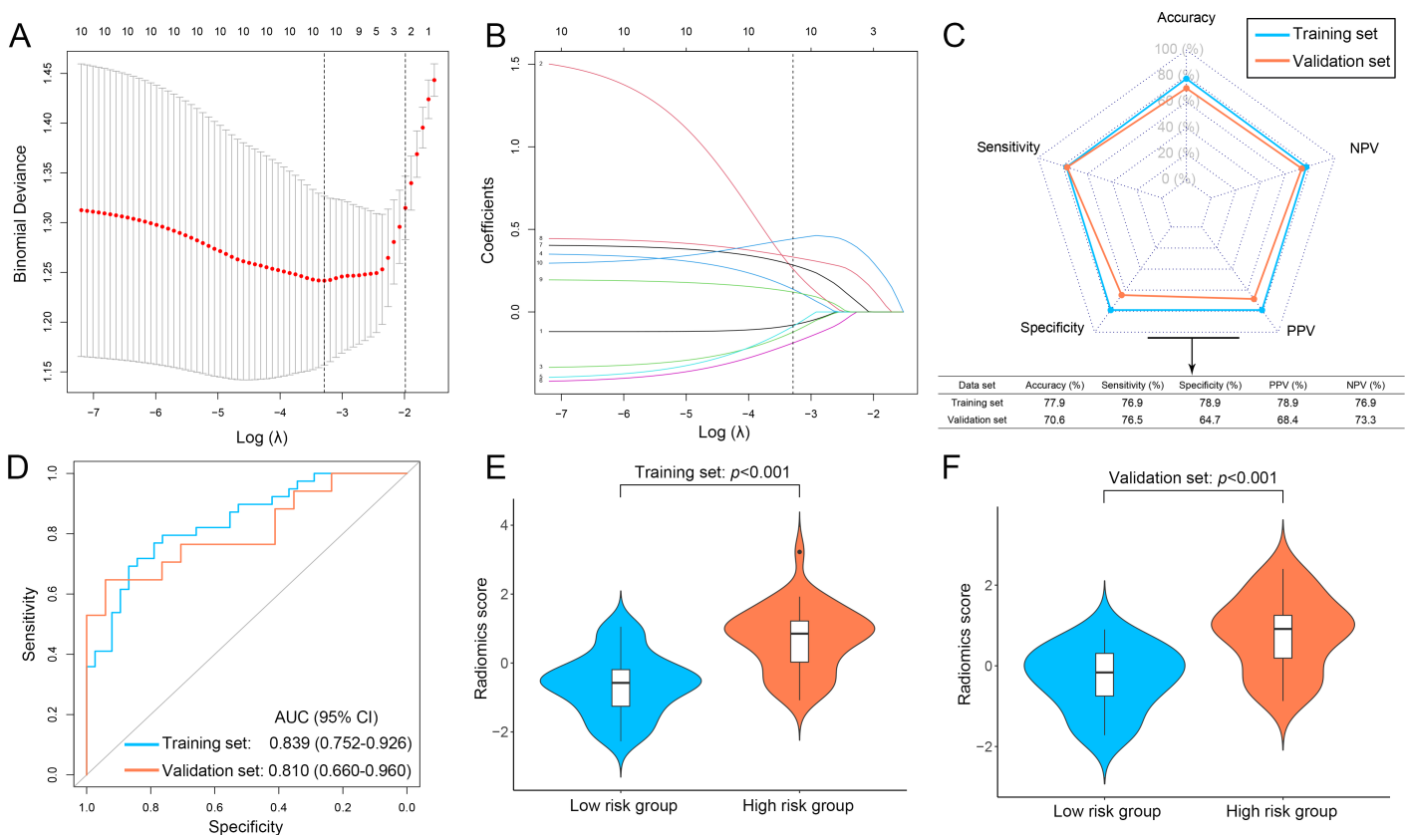
Development and performance of the radiomics signature. (**A**) Selecting the optimal number of features based on minimum criteria in the training set. (**B**) Based on the optimal λ value, 10 radiomics features were selected. (**C**) The performance of the radiomics signature in training and validation sets. (**D**) The ROC curves of the radiomics signature in training and validation sets. (**E**) Violin plot of the radiomics signature in the training set. (**F**) Violin plot of the radiomics signature in the validation set. NPV, negative predictive value; PPV, positive predict value; ROC, receiver operating curve; AUC, area under the ROC curve.

In addition, 0.762 to 0.908 was determined as the range of the inter-reader ICC between the two radiologists, indicating favorable inter- and intraobserver reproducibility. The features in the top 10 positions as ranked by mRMR were retained and utilized for the development of radiomics signatures. Therefore, we constructed a LASSO model based on these ten features (Figure 9A,B). These aforementioned features did not show a strong correlation with one another (Figure S1; mean absolute Spearman ρ = 0.158), revealing that the valuable radiomics features were obtained and that overfitting was avoided. The feature’s coefficients were also examined (Figure S1). The performance of the LASSO model was investigated (Figure 9C), with AUC values for the training and validation sets of 0.839 and 0.810, respectively (Figure 9D). In addition, individuals in the high-risk group showed considerably elevated radiomics scores as compared to those in the low-risk group in the two sets (Figure 9E,F; both *p* < 0.001).

## 4. Discussion

TIICs are vital constituents of non-tumor cells in TME, and the interaction between TIICs and tumor cells plays a vital role in tumorigenesis and malignant progression [24]. This research focused on investigating the different immune infiltrations and immune activities between molecular subtypes. The results showed that 11 out of 22 TIICs were significantly different between luminal and basal subtypes. This indicated that the dysregulation of TIICs could partly explain the distinct treatment responses and prognoses between luminal and basal subtypes. Previous research has reported the vital functions of macrophages in tumor angiogenesis, progression, metastasis, therapeutic resistance, and immune escape [25,26,27]. Neutrophils play an important role in the TME and tumor progression through secreting certain factors [28]. Thus, high infiltration of neutrophils may contribute to the malignant tumor behavior related to metastasis and poor prognosis in basal subtypes. Macrophages M1 promote antitumor immunity by secreting reactive oxygen species and proinflammatory cytokines, and activated NK cells play crucial roles in protective immunity against tumors [29]. Our results demonstrated that those in the basal subtype had significantly higher percentages of Macrophages M1 and activated NK cells than those in the luminal subtype, which revealed the high immune activity in basal subtypes. Conversely, the luminal subtypes had a higher percentage of regulatory T cells, which can inhibit tumor immune response. Thus, the results of our study showed an enrichment for immune infiltration and immune activity in basal subtypes, which was consistent with previous reports [30,31]. The molecular subtypes are associated with different TIICs and likely with different responses to immunotherapy, suggesting that the molecular subtypes should be considered for further clinical studies involving immunotherapy.

The molecular differences between molecular subtypes and immunotherapy response in BLCA comprise a complex multigenic process instead of a single gene functioning in isolation [8,13]. Currently, several immune-related models have been built for predicting prognoses or immunotherapeutic responses in BLCA [11,32,33,34]. However, most of these models have a single function, and the relationship between models and molecular subtypes has not been investigated. Thus, this research focused on developing a model based on multiple IRGs for molecular subtyping, prognostic prediction, and immunotherapy response prediction. A five-gene-based IPS was constructed that had a high association with molecular subtypes and critical immune checkpoint gene expression. The above-mentioned IPS demonstrated good performance in predicting the prognoses and immunotherapy responses of BLCA subjects. Specifically, IPS was negatively related to the expression profiles of critical immune checkpoint genes, so BLCA subjects in the low-risk group have high expression of critical immune checkpoint genes, thereby contributing to immunotherapy response and favorable prognosis. In addition, the capability of IPS to predict patient prognosis was further validated with several GEO data sets and comprehensively evaluated by means of a meta-analysis. Thus, the IPS is a multifunctional model and may be a beneficial tool for the evaluation of molecular subtyping, prognostic prediction, and treatment decision making. An IPS-based nomogram was constructed to be used by clinicians. The C-index and the calibration plots indicated that the 1- and 3-year OS prediction of the nomogram was in line with the actual 1- and 3-year OS. The clinical predictive superiority of this nomogram was established by DCA. Overall, the IPS and the IPS-based nomogram may be useful reference tools to assist in clinical decision making.

To further investigate the oncogenic mechanism and biological function of the IPS, GSEA was carried out for the IPS. Notably, the results revealed high enrichment levels of several oncogenic pathways in the high-IPS subgroup, which could explain the positive relationship between IPS and malignant clinicopathological features and poor prognoses.

Among the five genes in the IPS, CTLA4 is an important negative regulator of T cells [35] and can inhibit T cell function via various mechanisms [36,37], which contributed to the clinical development and application of anti-CTLA4 for tumor immunotherapy [38]. CD8A acts as a coreceptor with the T-cell receptors on T cells to recognize antigens displayed by antigen-presenting cells in the context of class I MHC molecules. Our previous study reported that CD8A is a novel indicator for predicting prognosis and immunotherapeutic response in BCa [39]. GNLY and FAM3B have been selected as hub genes in previous models for predicting prognosis and immunotherapy response in BCa [32,40], revealing the good performance of the two genes in prognostic and immunotherapy response prediction. CD3G is involved in T-cell development and signal transduction and has been reported to be associated with TME and prognosis in tumor patients [41,42]. However, no studies have investigated the role of CD3G in prognosis and TME in BCa.

In this study, we also tried to predict IPS scores through MRI-based radiomics in our clinical patient cohort. Notably, radiomics has already been identified as a feasible and effective method to predict tumor immune phenotypes, such as CD8+ T cell infiltration or neutrophil-to-lymphocyte ratios, even PD-1 treatment outcomes [43,44,45,46]. Using paired MRI and RNA sequencing, radiomics phenotypes were also found to be involved in immune regulation [47]. Therefore, it is possible to establish a radiomics model to preoperatively acquire tumor immune features. In bladder cancer, radiomics has been used to predict clinical pathological factors, such as prognosis, muscle-invasive status, and tumor grade, which could reduce the variability created by human error and provide more non-invasive information to guide treatment decisions [48,49]; however, few studies have used radiomics to predict tumor immune features in BLCA so far. This research utilized ten of the extracted radiomics features from DCE and T2WI images to construct a risk-predictive radiomics signature for BLCA subjects. The risk prediction for BLCA subjects was efficient in the validation and training sets, indicating the potential of some features of MRI-based radiomics to characterize the biological behavior at tumor onset. Notably, for this signature, the number of radiomics features from DCE and T2WI were both five, implying that the features of both have equal significance in the assessment of IPS.

This research has a few limitations. One is the absence of mechanistic analyses of the hub genes in BLCA, which need further functional studies in the future. Another limitation is the small number of BLCA subjects in our center. An increased number of BLCA subjects with RNA-sequence, preoperative MRI, and prognostic information are needed to further validate the prognostic value of the IPS and evaluate the performance of the radiomics signature in IPS prediction.

## 5. Conclusions

This research indicated that half of the TIICs between luminal and basal subtypes were different. The five-gene IPS had the possibility of serving as a clinically promising model for molecular subtyping, prognostic prediction, and immunotherapy response prediction. GSEA analysis clarified the biological function of the IPS in the tumor progression of BLCA. An MRI-based radiomics signature can preoperatively assist in predicting patients’ IPSs, thereby contributing to the preoperative prediction of prognosis and immunotherapeutic susceptibility in BLCA.

## Figures and Tables

**Table 1:** Univariate and multivariable Cox regression analyses of the IPS clinicopathological features in The Cancer Genome Atlas.

	Univariate Analysis		Multivariate Analysis	
Variables	HR (95% CI)	*p*-Value	HR (95% CI)	*p*-Value
Age	1.03 (1.02–1.05)	<0.001	1.04 (1.02–1.05)	<0.001
Sex	1.19 (0.86–1.64)	0.308	-	-
Stage	1.74 (1.43–2.11)	<0.001	-	-
Grade	2.89 (0.72–11.68)	0.136	-	-
T stage	1.65 (1.33–2.04)	<0.001	1.37 (1.05–1.77)	0.018
M stage	2.64 (1.29–5.38)	0.009	-	-
N stage	1.56 (1.33–1.84)	<0.001	1.44 (1.20–1.72)	<0.001
Morphology	0.68 (0.48–0.97)	0.031	-	-
IPS	1.48 (1.20–1.83)	<0.001	1.33 (1.06–1.66)	0.015

HR, hazard ratio; IPS, immune prognostic signature; CI, confidence interval.

**Table 2:** Clinicopathological characteristics of patients.

Characteristic	Number of Patients (%)		*p*-Value
	Training Set (*n* = 77)	Validation Set (*n* = 34)	
Sex			
Men	66 (85.7)	25 (73.5)	0.124
Women	11 (14.3)	9 (26.5)	
Age (years)			
<65	22 (28.6)	11 (32.4)	0.688
≥65	55 (71.4)	23 (67.6)	
Tumor size (cm)			
<3	40 (51.9)	15 (44.1)	0.447
≥3	37 (48.1)	19 (55.9)	
Number of tumors			
Single	51 (66.2)	24 (70.6)	0.651
Multiple	26 (33.8)	10 (29.4)	
Pathological grade			
Low-grade	15 (19.5)	9 (26.5)	0.410
High-grade	62 (80.5)	25 (73.5)	
Clinical T stage			
<T2	49 (63.6)	24 (70.6)	0.477
≥T2	28 (36.4)	10 (29.4)	

## Data Availability

The data sets used and/or analyzed during the current study are available from the corresponding author on reasonable request.

## Supplementary Materials

The following supporting information can be downloaded at: https://www.mdpi.com/article/10.3390/bioengineering10030318/s1, Figure S1. The coefficients of 10 features in the LASSO model.

## Abbreviations

BLCA	Bladder cancer
TCGA	The Cancer Genome Atlas
TIIC	Tumor infiltrating immune cell
IPS	Immune prognostic signature
GSEA	Functional enrichment analysis
LASSO	Least absolute shrinkage and selection operator
AUC	Area under the curve
NMIBC	Non-muscle invasive bladder cancer
MIBC	Muscle-invasive bladder cancer
IRGs	Immune-related genes
OS	Overall survival
PR	Partial response
CR	Complete response
GEO	Gene Expression Omnibus
mRMR	Minimum redundancy maximum relevance
ICCs	Intra- and interclass correlation coefficients
PPV	Positive predictive value
NPV	Negative predictive value
CI	Confidence interval
MRI	Magnetic resonance imaging
DCE	Dynamic contrast-enhanced
