# Supplementary material for: Development of a Molecular-Subtype-Associated Immune Prognostic Signature That Can Be Recognized by MRI Radiomics Features in Bladder Cancer
Source: Bioengineering (Basel). 2023 Mar 2;10(3):318. doi: 10.3390/bioengineering10030318 (PMC10045524; doi:10.3390/bioengineering10030318)
Supplement: Supplementary file 1 [file bioengineering-10-00318-s001.zip › bioengineering-2052510-supplementary.pdf]

Supplementary Information

Development of a molecular subtype-associated immune prognostic signature that can be recognized by MRI radiomics features in bladder cancer

Shenghua Liu[1, †], Haotian Chen[1,2, †], Zongtai Zheng[1,2], Yanyan He[3, *], Xudong Yao[1,2, *]

[1]Department of Urology, Shanghai Tenth People's Hospital, Tongji University, Shanghai, PR China;
[2]Urologic Cancer Institute, School of Medicine, Tongji University, Shanghai, China;
[3]Department of Pathology, Shanghai Tenth People's Hospital, Tongji University, Shanghai, PR China
† These authors contributed equally to this work.
* Correspondence: yaoxudong1967@163.com/ drfelixliu@163.com

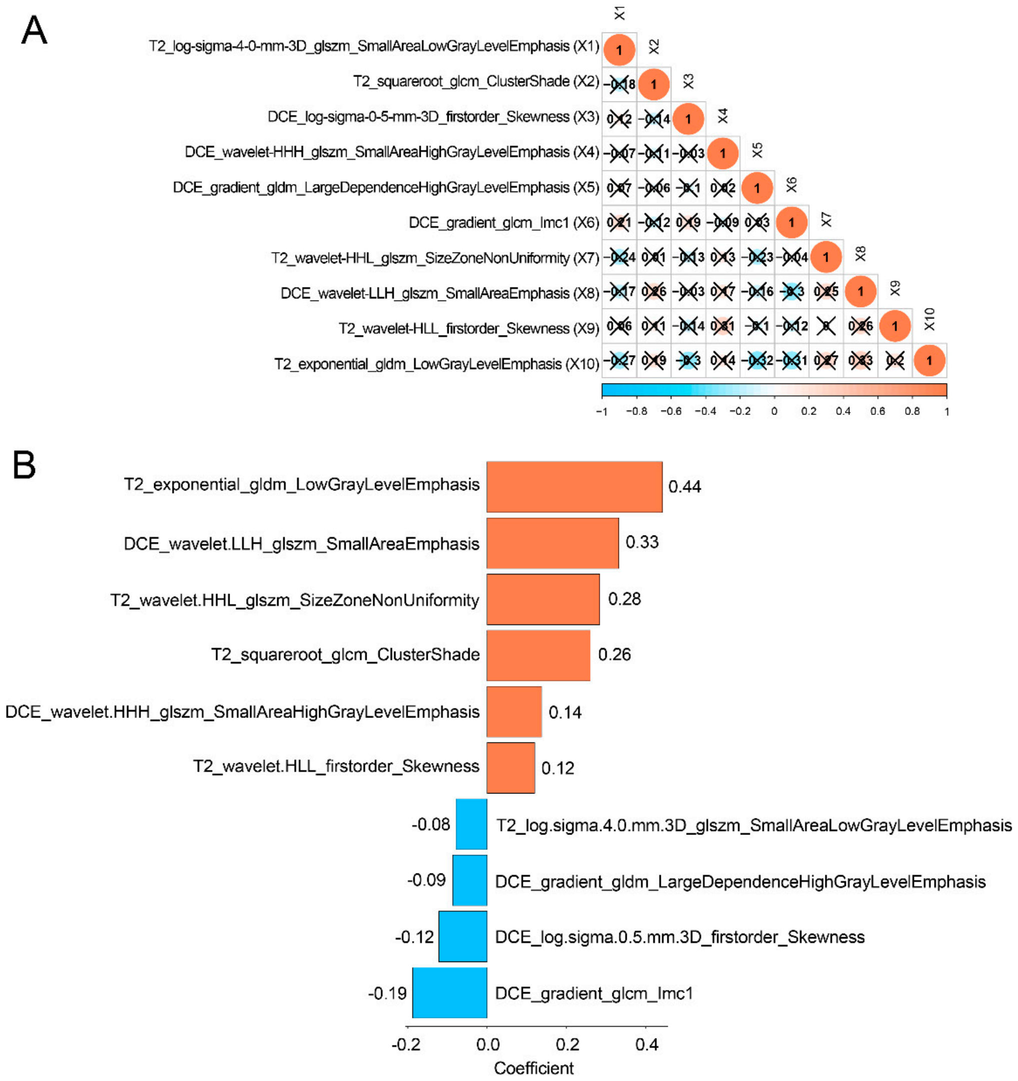

Figure S1. (A) The 10 features in the LASSO model were not highly correlated with each other (mean absolute Spearman ϱ = 0.158). (B) Coefficients of 10 Features in LASSO Model.
